# A comprehensive analysis of dry eye disease clinical trials (2000–2024): research trends and gaps

**DOI:** 10.3389/fphar.2026.1713433

**Published:** 2026-01-14

**Authors:** Xiaohui Jiang, Boyue Xu, Ruxin Xu, Nanxin Wu, Shudong Tian, Haotian Peng, Yun-e Zhao

**Affiliations:** 1 Eye Hospital and School of Ophthalmology and Optometry, Wenzhou Medical University, Wenzhou, China; 2 National Clinical Research Centre for Ocular Diseases, Wenzhou, China

**Keywords:** clinical trials, dry eye disease, geographical distribution, research trends, trial design

## Abstract

**Introduction:**

Dry eye disease (DED), also known as keratoconjunctivitis sicca (KCS), imposes a substantial global burden, yet the clinical trial landscape guiding therapeutic development remains incompletely characterized.

**Methods:**

We conducted a cross-sectional study mapping interventional dry eye disease trials (2000–2024) indexed in Trialtrove. We analyzed 1,171 prospectively registered trials with dry eye disease as the primary indication, characterizing design, phase, geography, sponsorship, endpoints, and therapeutics.

**Results:**

Trial activity rose steadily, peaking in 2023–2024, and was concentrated in the United States (n=773), followed by India (n=111), China (n=71), and Japan (n=58). Sponsorship was balanced between industry (45.5%) and academia (44.4%). Most trials were completed and in later phases (Phase 4: 40.3%; Phase 2: 25.0%; Phase 3: 17.0%). Adult cohorts predominated, yet 59.9% did not report severity strata, and where strata were prespecified, thresholds based on symptom questionnaires were heterogeneous. Monotherapy designs were most common (71.6%), with head-to-head comparisons in 22.4%. Frequently studied interventions included other ocular medications, ocular anti-inflammatory agents, and artificial tears. Primary endpoints centered on ocular surface staining, with the Ocular Surface Disease Index, tear film break-up time, and Schirmer’s test commonly used.

**Discussion:**

These commonly used endpoints vary in psychometric validity and standardization, which can undermine data quality, complicate analysis and interpretation of individual trial results, and limit cross-trial comparability. Overall, the ecosystem appears mature but conservative, emphasizing later-phase, single-agent studies, traditional endpoints, and limited severity stratification and geographic diversity. Future trials should prespecify severity and subtype strata, use validated mechanism-aligned endpoints with standardized assessment windows and longer follow-up, and broaden geographic representation to improve generalizability and cross-trial comparability.

## 1 Introduction

Dry eye disease (DED), historically referred to as keratoconjunctivitis sicca (KCS), dry eye syndrome (DES), or dysfunctional tear syndrome, is a common ocular surface disorder characterized by tear film instability and symptoms such as discomfort and visual disturbance. It can lead to ocular surface damage and significantly impair quality of life (Eye WorkShop, 2007). Dry eye disease not only impairs physical health but also significantly impacts mental wellbeing, with studies showing that patients with dry eye symptoms have 1.47 times higher odds of suicidal ideation (Um et al., 2018; Kim et al., 2023). Global studies report a prevalence of DED ranging from 5% to 50% in adults over 30 years old, with higher rates in Asia and among older adults. In some Asian cohorts aged over 60 years, prevalence exceeds 30%, and women are affected more frequently than men (Schaumberg et al., 2003; Farrand et al., 2017; Song et al., 2018; Caffery et al., 2019). Utility studies indicate that severe DED can be perceived as comparable to severe angina or dialysis-level health states (Schiffman et al., 2003; Buchholz et al., 2006). The direct medical burden is substantial; in the United States, annual costs reach approximately $3.8 billion (Yu et al., 2011). Despite diverse available therapies, many patients experience persistent symptoms or recurrent flares, reflecting the clinical heterogeneity and limitations of current treatment approaches (Messmer, 2015; Marshall and Roach, 2016; Dogru et al., 2013).

Consequently, clinical research in DED has expanded markedly in recent years, aiming to deepen the understanding of disease mechanisms, identify novel therapeutic targets, and address unmet clinical needs (Stahl et al., 2006; De Paiva et al., 2006; Sonawane et al., 2012; Tibrewal et al., 2013). However, although the number of registered clinical trials continues to grow worldwide, there remains a notable gap in integrated analyses that systematically examine trial characteristics such as design, phase, sponsor type, geography, outcome and endpoint selection, and that track their evolution over time. Existing studies are often confined to specific regions or intervention types, and no coordinated effort has been made to synthesize global trends within a unified empirical framework (Messmer, 2015; Dogru et al., 2013).

To address this gap, we conducted a registry-based overview of interventional DED trials indexed in Trialtrove from 2000 through 2024. We characterized trials by design class, phase, sponsor type, geography, and patient segments. Within monotherapy studies, we summarized drug classes and organized outcomes into endpoint families commonly used in DED research. We also identified important limitations in the design and reporting of existing DED clinical trials, including incomplete severity stratification, heterogeneous outcome measures, and variable procedural standardization. By synthesizing this information, we aim to provide a comprehensive landscape of current research efforts, thereby informing the design of future clinical studies and supporting the development of innovative therapies for dry eye disease.

## 2 Materials and methods

### 2.1 Study design, data source, and search strategy

This cross-sectional study analyzed prospectively registered interventional clinical trials on dry eye disease (DED) indexed in Trialtrove (Citeline, Informa Pharma Intelligence) (https://clinicalintelligence.citeline.com/), a globally curated clinical trials database with standardized aggregation and validation processes (Wu et al., 2022; Kammula et al., 2024). Trialtrove routinely integrates and harmonizes records from major primary registries (e.g., ClinicalTrials.gov, EU Clinical Trials Register) and national authorities to provide comprehensive, high-quality coverage of clinical research activity worldwide. We comprehensively retrieved all registered interventional trials whose registry records contained references to “dry eye,” “dry eye disease” and “keratoconjunctivitis sicca,” using platform-specific queries designed to capture all such trials. The retrieval window covered January 2000 through December 2024. All records identified within this period were exported in full for analysis. Trials indexed in Trialtrove without public registration identifiers were retained and labeled “Unregistered.”

### 2.2 Eligibility criteria, study selection, and data extraction

We included interventional trials enrolling participants with DED as the primary indication, irrespective of phase, sponsor type, or geography. We excluded observational studies, expanded-access programs, preclinical/animal/*in vitro* investigations, duplicate records, withdrawn entries lacking substantive protocol information, and records with incomplete essential fields after cross-checking. Two investigators independently screened titles and registry summaries; discrepancies were resolved by a third investigator. Using a predefined data dictionary, we extracted trial identifiers and dates; design features (phase, masking, allocation, interventional model, and design class); geography; sponsor/collaborator type; participant eligibility (age band, sex); disease severity strata; interventions (agent names, routes, and drug classes); and outcomes (primary and secondary endpoints, timing). Sponsorship was determined by the primary sponsor listed in Trialtrove and classified as industry (pharmaceutical or device companies) or academia (universities, hospitals, research institutes, foundations, or public agencies). Trials with an industry primary sponsor were considered industry-sponsored, regardless of study site, whereas those with an academic or public primary sponsor were considered academic, regardless of any industry collaboration (e.g., drug supply or partial funding). For patient-reported outcome measures (PROMs; e.g., OSDI, SPEED), we recorded the instrument name/version/language and whether validation/psychometric sources were cited in the registry; when absent, items were coded as ‘unspecified.’ Primary versus secondary outcomes were distinguished using registry-designated fields (e.g., “Primary Outcome Measure”). For ocular surface staining we sought, when available, the dye and delivery method (for example fluorescein or lissamine and strip versus microdrop), the observation window and illumination or filter, the grading scale (for example NEI or Industry, Oxford, van Bijsterveld), the reading model (site based versus central or dual masked), any site training or certification, and investigator subspecialty. When this information was not available, we coded the item as “unspecified.”

### 2.3 Definitions and classification rules

Definitions and classification rules were prespecified to ensure reproducibility: (1) trial design (monotherapy, head-to-head comparisons, combination drug therapy, multimodal therapy, non-pharmacological interventions, other); (2) drug classes for monotherapy (ocular anti-inflammatory agents, artificial tears, secretagogues, ocular surface repair agents, ocular/systemic antibiotics, systemic non-antibiotic medications, other ocular medications); (3) endpoint families (ocular surface staining, OSDI, TBUT, Schirmer’s test, VAS-based symptom scores), prioritized by registry-defined primary vs. secondary designation; (4) non-ophthalmology therapeutic co-labels when present alongside DED in the registry. Classifications were derived from registry metadata, agent labels, ingredient names, approved indications. VAS based symptom scores were grouped as one endpoint family. When registry text specified a 0–100 format, this was recorded; otherwise format and recall period were treated as not reported and no imputation was performed. Neurosensory pain refers to ocular surface pain arising from abnormal peripheral nociception and/or central sensitization, often manifesting as burning, dysesthesia, allodynia, or hyperalgesia and sometimes occurring with minimal observable corneal staining (Spierer et al., 2016; Kolk et al., 2002).

### 2.4 Statistical analysis

Categorical variables were summarized as counts and percentages. Unless otherwise stated, the denominator for proportions was the total number of eligible trials; for monotherapy drug classes, the denominator was the monotherapy subset. Visualizations (line charts, bar plots) were used to illustrate annual patterns and geographic distributions. Continuous variables, where applicable, were summarized as medians with interquartile ranges or means with standard deviations. All statistical analyses and graph plotting were performed using R version 4.3.0 (R Foundation for Statistical Computing, Vienna, Austria) and GraphPad Prism version 8.0.2 (GraphPad Software, Boston, MA).

### 2.5 Ethics approval and consent to participate

This study utilized publicly available secondary data from Trialtrove database, requiring no institutional review board approval or consent. This study adheres to the STROBE guidelines for observational studies to ensure transparency and methodological rigor.

### 2.6 Availability of data and materials

The original contributions presented in the study are included in the article/supplementary material, further inquiries can be directed to the corresponding author/s.

## 3 Results

### 3.1 Sustained growth in global trial activity with persistent U.S. leadership

**FIGURE 1:**
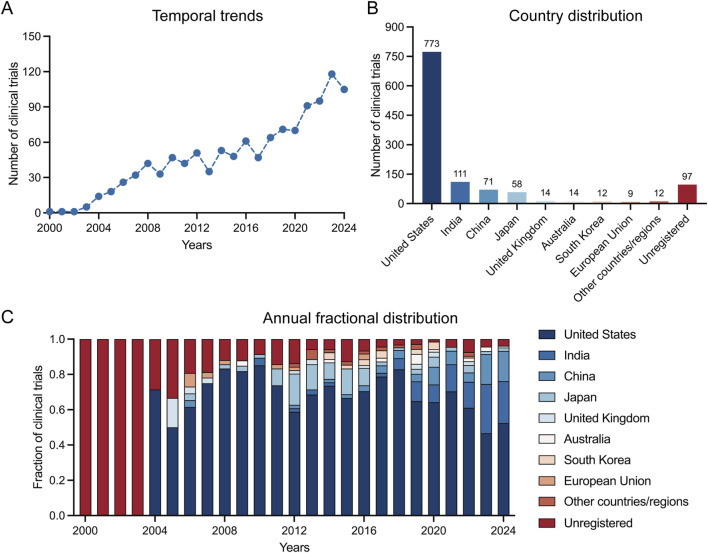
Temporal and geographic distribution of dry eye disease clinical trials. **(A)** Annual number of trials. **(B)** Country/Region distribution; “Unregistered” denotes studies indexed in Trialtrove without public registration identifiers. **(C)** Annual fractional contribution of countries/regions (each bar sums to 100% of trials initiated that year).

Global DED clinical trial activity increased from 2000 to 2024, with the annual number of trials rising steadily to a recent peak in 2023 and 2024 (Figure 1A). While trial activity was global, it was predominantly concentrated in the United States (n = 773), which was followed distantly by India (n = 111), China (n = 71) and Japan (n = 58). The United Kingdom (n = 14), Australia (n = 14), South Korea (n = 12), and the European Union (aggregate n = 9) contributed smaller proportions, and 97 trials were unregistered (Figure 1B). Analysis of year-by-year researches reveals that although the U.S. consistently held the largest share, overall participation diversified over time with increasing contributions from Asia-Pacific countries (Figure 1C).

### 3.2 Majority of completed, late - phase trials in broad adult populations

**FIGURE 2:**
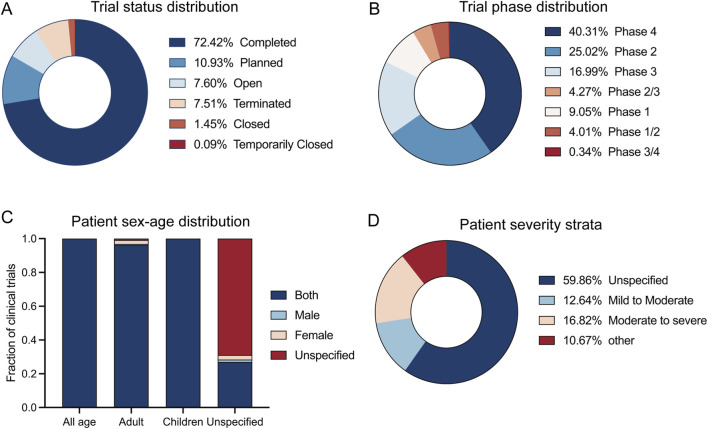
Trial status, phase, and participant characteristics. **(A)** Distribution of trial status. **(B)** Distribution of trial phases. **(C)** Eligibility by age group and sex; “Unspecified” indicates no restriction on age and/or sex in the inclusion criteria. **(D)** Patient severity strata; “Unspecified” indicates that the registry record did not report a severity classification.

At the time of data freeze, most trials were completed (72.42%), while 10.93% were planned, and 7.60% were open. Smaller proportions were terminated (7.51%), closed (1.45%), or temporarily closed (0.09%) (Figure 2A). Later-phase studies predominated: Phase 4 trials constituted the largest share (40.31%), followed by Phase 2 (25.02%) and Phase 3 (16.99%). Phase 1 accounted for 9.05%, with combined phases (Phase 1/2 and 2/3) comprising 4.01% and 4.27%, respectively; Phase 3/4 trials were rare (0.34%) (Figure 2B). The vast majority of trials included adult participants of both sexes, with single-sex and pediatric-only trials being uncommon (Figure 2C). Regarding disease severity, a majority (59.86%) did not specify a stratum. Among those that did, moderate-to-severe disease was the most common specified category (16.82%), followed by mild-to-moderate (12.64%) and other specific segments (10.67%) (Figure 2D).

### 3.3 Endpoints, sponsorship, and non-ophthalmology therapeutic associations

**FIGURE 3:**
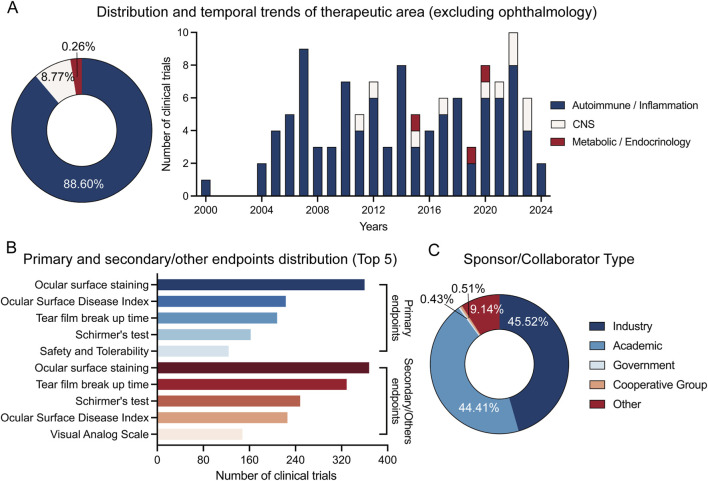
Non-ophthalmology therapeutic sub-areas, endpoints, and sponsor types. **(A)** Therapeutic areas outside ophthalmology: annual counts for non-ophthalmology subareas: Autoimmune/Inflammation (n = 101, 88.60%), Central Nervous System (n = 10, 8.77%), and Metabolic/Endocrinology (n = 3, 0.26%). All trials are ophthalmology by indication. **(B)** Distribution of the top five primary and secondary/other endpoints. **(C)** Sponsor/Collaborator types across all trials.

A small subset of trials were additionally classified outside ophthalmology, chiefly within Autoimmune/Inflammation (88.60% of the non-ophthalmology subset), with fewer in Central Nervous System (8.77%) and Metabolic/Endocrinology (0.26%). Temporal bars show intermittent but persistent activity across these sub-areas from 2000 onwards (Figure 3A). Endpoint selection converged on established measures. PROMs most commonly included OSDI and SPEED. Contemporary evidence indicates good internal consistency but multidimensionality and variable Rasch-based properties for these instruments, limiting interpretability of single summary scores; registry entries rarely cited validation sources, precluding appraisal of PROM quality (Baral et al., 2025). As primary endpoints, ocular surface staining was most frequently used (approximately the leading category), followed by the Ocular Surface Disease Index, tear film break-up time, and Schirmer’s test. The same families of outcomes dominated secondary/other endpoints, with ocular surface staining again ranking first, followed by tear film break-up time, Schirmer’s test, OSDI, and visual analog scale–based symptom scores (Figure 3B). Sponsorship was balanced between industry (45.52%) and academic bodies (44.41%), with smaller shares from government (0.51%) and cooperative groups (9.14%) (Figure 3C). Registry records seldom reported the number of points or the recall period for symptom scales. When specified, VAS was most often recorded on a 0–100 range.

Among trials that prespecified disease severity strata, criteria were predominantly symptom-based and heterogeneous. Most relied on OSDI thresholds, but cut points varied (for example OSDI≥23 for moderate-to-severe and ≥33 for severe; some divided OSDI into 0–12, 13–22, 23–32, and 33–100 bands). Others used SANDE (for example ≥50 for moderate-to-severe or 20–55 for mild-to-moderate), DEQ-5 (for example ≥6 as screen-positive and ≥12 as severe), or SPEED scores, with objective signs incorporated inconsistently.

### 3.4 Trial design classes and drug categories among monotherapy studies

**FIGURE 4:**
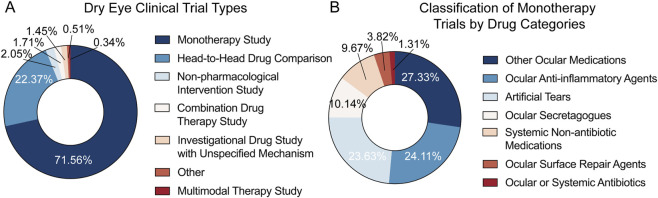
Trial design types and monotherapy drug classes. **(A)** Classification of trial design types. **(B)** Distribution of drug classes among monotherapy trials (subset defined in **(A)**).

Monotherapy constituted the majority of DED trial designs (71.56%), followed by head-to-head drug comparisons (22.37%). Non-pharmacological interventions (2.05%), combination drug therapy (1.71%), investigational drugs with unspecified mechanism (1.45%), “other” designs (0.51%), and multimodal therapy (0.34%) were comparatively uncommon (Figure 4A). Among monotherapy studies (n = 853), sponsorship was industry 50.76%, academic bodies 45.37%, government 0.82%, cooperative groups 1.06%, and other 1.99%, and trials were predominantly later-stage, with 16.76% in Phase III, 0.35% in Phase III/IV, and 38.45% in Phase IV. Drug classes were distributed across “other ocular medications” (27.33%), ocular anti-inflammatory agents (24.11%), artificial tears (23.63%), ocular secretagogues (10.14%), systemic non-antibiotic medications (9.67%), ocular surface repair agents (3.82%), and ocular or systemic antibiotics (1.31%) (Figure 4B). Collectively, these distributions indicate a portfolio dominated by single-agent evaluations and anchored in established ocular therapies, with a smaller but notable representation of systemic and repair-focused approaches.

## 4 Discussion

Leveraging Trialtrove registry data (2000–2024), this study maps interventional trials in DED across geography, phase, design, outcomes, and interventions. Trial volume rose steadily, peaking recently; the United States remained dominant, with growing Asia–Pacific contributions (notably India, China, Japan). Later-phase and single-agent studies predominated, while head-to-head and multimodal trials were scarce. Severity reporting was often missing, and outcomes clustered around the traditional quartet corneal/conjunctival staining, OSDI, TBUT, and Schirmer’s test. Single-agent studies were mainly “other ophthalmic agents,” anti-inflammatories, and artificial tears, with relatively few targeting evaporative pathophysiology and lipid-layer stability. Overall, a maturing yet conservative ecosystem limits cross-trial comparability and strategy-grade inference.

### 4.1 Geographic distribution and external validity

The sustained US dominance in trial conduct, together with the recent rise in Asia Pacific activity, raises practical questions about external validity and regional adaptation. Climatic conditions, environmental exposures, and healthcare delivery patterns shape DED expression and response heterogeneity. Evidence generated within specific North American trial networks may not fully generalize to regions with different environmental profiles, including monsoon and subtropical climates. These concerns also apply to populations with distinct risk profiles and digital device use patterns, which is particularly salient in Asia (Talens-Estarelles et al., 2023; Talens-Estarelles et al., 2021). Our dataset shows notable participation growth in India, China, and Japan; however, the absolute base remains substantially lower than in the United States, and later-phase trials continue to cluster in US-centric networks, potentially constraining Asia Pacific–specific insights into dosing, adherence, and acceptability. Increasing the proportion of Asia Pacific participants in confirmatory studies and embedding region-specific substudies would enable calibration of symptomatic and tear-film outcomes to local environmental contexts, while also strengthening health-technology assessment applicability. Prior reviews emphasize population differences and modifying factors, including sex and hormonal influences, that intersect with geography. These insights support intentional sampling across regions and demographic strata to improve generalizability (Cutrupi et al., 2023; Akpek et al., 2019; Wróbel-Dudzińska et al., 2023; Barabino et al., 2020).

### 4.2 Trial design and endpoint selection

The analysis found that 59.9% of trials did not report severity strata. Among those that did, cohorts skewed toward moderate to severe disease. Even when patients are stratified, heterogeneity in PROM instruments and cut points (for example variable OSDI bands and SANDE, DEQ 5, and SPEED thresholds) raises concerns about misclassification. The observed thresholds (for example OSDI ≥23 vs. ≥33 to define similar categories, and differing SANDE and DEQ-5 bands) illustrate the lack of standardization and the predominantly symptom-based nature of severity definitions, with objective signs incorporated inconsistently. Consistent with Baral et al., limitations in PROM development and content validity further challenge the accuracy of symptom based categories. When strata were omitted in 59.9% of trials or applied inconsistently, selection and analytical bias were likely, which compromised data quality and warranted cautious interpretation of both individual trial results and pooled effects (Baral et al., 2025). Outcomes clustered on ocular surface staining, OSDI, TBUT, and Schirmer’s test. Ocular surface staining, TBUT, and Schirmer tests are operator and protocol dependent, and dye and fluorescein load and delivery, timing and illumination, grading and scoring, environmental factors, Schirmer type, use of topical anesthetic, strip placement and duration, and when NIBUT is used device choice and calibration all introduce variability, so equal reading skills across sites should not be assumed. Multicenter trials should prespecify SOPs, require site training and certification, maintain calibration logs, and use central or dual masked reading where feasible (Savini et al., 2008).

The quality of commonly used PROMs is heterogeneous: OSDI and SPEED are widely used yet multidimensional with variable targeting/responsiveness, which can obscure construct-specific change. Trials should preferentially select PROMs with stronger psychometric evidence (Rasch-calibrated where available) and report instrument version/language, scoring, and minimally important difference (Baral et al., 2025). In DED trials, the OSDI warrants cautious interpretation despite its widespread use. Developed in an industry setting with unrestricted Allergan support (Schiffman et al., 2000), it uses a 1 week recall, frequency based responses, and a single composite score merging symptoms, vision related function, and environmental triggers. Psychometric evaluations, including Rasch analyses, indicate multidimensionality, suboptimal targeting, and only fair precision, with limited patient led content by contemporary standards, and correlations with objective signs are generally weak outside select subgroups (Baral et al., 2025; Schiffman et al., 2000). These limitations do not negate OSDI’s internal consistency and known group discrimination, but they constrain cross trial comparability and may bias severity classification when frequency substitutes for intensity. Accordingly, we recommend prespecifying PROM choice and recall windows, reporting domain level scores when multidimensional, considering Rasch calibrated or item bank instruments, and pairing symptom PROMs with mechanism aligned signs (Baral et al., 2025).

Because patients prioritize symptom relief, symptom severity is a critical outcome dimension; protocols should prespecify symptom-severity-based eligibility, baseline stratification, and responder analyses anchored to minimally clinically important difference (MCID) and patient-acceptable symptom state (PASS), where available. Symptom scales should be prespecified with explicit format, anchor wording, recall period, and choice of metric. Although VAS are common for symptom reporting, psychometric work and PROMIS guidance support transitioning to standardized 11 point numeric rating scales for symptom intensity (Galor et al., 2018). These scales provide more precise and interpretable estimates of symptom intensity and enhance cross trial comparability, and can be implemented using validated instruments such as the PROMIS Pain Intensity short form or similar item bank based tools. A consistent 7 days recall window is commonly used in PROMIS for chronic symptoms, whereas a current or 24 h window may be more appropriate for acute effects. Site training and centralized guidance should be provided to ensure uniform administration across centers.

Sensitivity and timing differ between symptoms and signs. Prior clinical overviews report variable and often weak correlations between signs and symptoms in dry eye disease and emphasize the heterogeneity of mechanisms and frequent comorbidities. The sign–symptom gap reflects multiple factors, including measurement limitations, disease heterogeneity, and variability in clinical evaluation and comorbidity recognition, and is not confined to neurosensory or inflammatory mechanisms. Accordingly, diagnosis and monitoring benefit from a mechanism-aligned, multidimensional assessment that integrates validated patient-reported outcomes, tear-film stability and surface staining patterns, meibomian gland function, and context-specific inflammatory and neurosensory evaluations. Given the non-specificity of isolated markers such as tear hyperosmolarity and a simple presence/absence of inflammation, we emphasize a specific, mechanism-aligned composite that includes validated symptom measures, tear breakup time and pattern, corneal and conjunctival staining distributions, meibomian gland structure and expressibility, and targeted evaluation for ocular and systemic comorbidities known to modulate pain and inflammation (Spierer et al., 2016; Kolk et al., 2002; Baral et al., 2025; Nichols et al., 2004; Sullivan et al., 2014; Vehof et al., 2017; Wolffsohn et al., 2017; Bron et al., 2017).

For evaporative disease, endpoints should include interferometric lipid metrics, evaporation rate, tear film stability, and key obstructive meibomian gland dysfunction measures such as lid tenderness on gentle palpation, meibomian gland expressibility with meibum quality, lid margin signs including orifice plugging and telangiectasia with mucocutaneous junction displacement, noninvasive tear breakup time with breakup pattern, blink rate with partial blink ratio, and meibography derived gland dropout scores. Omission of lid tenderness and gland expressibility should be noted as a protocol deficiency (Wolffsohn et al., 2017; Nichols, 2011; Mask et al., 2019; Huang et al., 2019). For aqueous deficient disease, endpoints include Schirmer testing with or without anesthesia, phenol red thread, tear meniscus height or area by slit lamp or OCT, corneal and conjunctival staining such as the NEI grid, optional impression cytology for goblet cell density, and context specific biomarkers such as tear osmolarity and MMP-9 acknowledging limited specificity (Wolffsohn et al., 2017; Willcox et al., 2017). Primary endpoints should align with dominant mechanisms. Symptom-sign composites should be retained with hierarchical testing to safeguard interpretability (Savini et al., 2008; Wolffsohn et al., 2017). Assessment windows should be standardized beyond early readouts. Mid to long term time points are needed to capture maintenance effects, which were under assessed in many studies. This approach also addresses the high overlap between primary and secondary outcome families and the commonly short follow up, thereby improving cross trial comparability and synthesis.

### 4.3 Therapeutic approaches and mechanistic gaps

Given the multifactorial nature of dry eye disease and the heterogeneity of ocular surface comorbidities, persistent methodological limitations in many clinical trials, including reliance on nonspecific or imperfect endpoints, lack of prospective subtype stratification, and inconsistent assessment methods and assessment windows, contribute to suboptimal patient outcomes and overestimation of treatment effects. Future trial portfolios should prioritize mechanism-specific interventions while maintaining neutrality about efficacy. Approaches currently under investigation, such as strategies that address both inflammatory and neurosensory pathways, regenerative medicine modalities that leverage paracrine activity, and agents that aim to support ocular surface barrier function, are examples of mechanism-aligned hypotheses that require rigorous evaluation. Robust appraisal depends on prespecified, validated, mechanism-aligned endpoints and prospectively defined patient subgroups, with standardized assessment schedules and transparent reporting. These recommendations are intended to guide future protocols and the interpretation of ongoing studies rather than imply mid-course changes or endorse any specific therapy (Moawad et al., 2022; Anitua et al., 2015; Fini et al., 2016).

### 4.4 Limitations

This registry-based analysis has several limitations. While Trialtrove aggregates data from multiple global registries, there may be a lag in indexing smaller regional or non-English language registries compared to major international platforms, which could introduce geographical selection bias and potentially underrepresent trials conducted in certain developing regions. Although reliance on the Trialtrove database provides broad coverage of DED trials, under-representation of certain regions, particularly developing countries, may limit generalizability. We may also have missed unpublished or unregistered studies, introducing potential selection bias. In addition, our findings depend on registry entries that were sometimes incomplete or inconsistently reported, especially for study endpoints, patient demographics and subgroups, and symptom scale characteristics (such as scale format and recall period), which may affect classification accuracy. Registry data lacked investigator-level conflict-of-interest disclosures, precluding assessment of disclosure rates across trials. Key procedural details were rarely specified, including ocular surface staining execution, TBUT procedures, Schirmer protocol elements (type, anesthetic use, strip placement and duration), reading models, site training and certification, and investigator subspecialty, limiting our ability to evaluate uniformity across sites. These factors should be considered when interpreting our results and when designing future registry-based analyses. Additionally, Trialtrove is a subscription-based platform, which may limit independent verification by researchers without institutional access. The lack of standardized age reporting across registries also limited age-stratified analyses, particularly for elderly populations.

## Data Availability

The original contributions presented in the study are included in the article/supplementary material, further inquiries can be directed to the corresponding author.
